# Moderate-to-severe atopic dermatitis and systemic corticosteroids are associated with insulin resistance and metabolic syndrome

**DOI:** 10.1016/j.jdin.2025.09.021

**Published:** 2025-10-25

**Authors:** Emilio Berna-Rico, Bibiana Pérez-Garcia, Enrique Gómez-de la Fuente, Javier Pérez-Bootello, Carlota Abbad-Jaime de Aragon, Fernando Neria, Diana Monge, Raquel Domínguez-López, Carlos J. Aranda, Pedro Jaen, Nehal N. Mehta, Joel M. Gelfand, Andrew Blauvelt, Álvaro González-Cantero

**Affiliations:** a Department of Dermatology, Hospital Universitario Ramón y Cajal, IRYCIS, Madrid, Spain; b Facultad de Medicina, Universidad Francisco de Vitoria, Madrid, Spain; c Allergy Unit and Research Group, Hospital Regional Universitario de Málaga and IBIMA-Plataforma BIONAND, RICORS Inflammatory Diseases, Málaga, Spain; d Department of Cardiology, George Washington Medical Center, Washington, District of Columbia; e Department of Dermatology and Center for Clinical Sciences in Dermatology, University of Pennsylvania Perelman School of Medicine, Philadelphia, Pennsylvania; f Blauvelt Consulting, LLC, Portland, Oregon

**Keywords:** atopic dermatitis, atopic eczema, comorbidities, corticosteroids, eczema, insulin resistance, metabolic syndrome

*To the Editor:* Recent studies have re-defined the understanding of atopic dermatitis (AD) as a systemic inflammatory disease, extending beyond its well-established associations with allergic conditions to include potential cardiovascular co-morbidities.^1^ The relationship between AD, insulin resistance (IR), and metabolic syndrome (MetS), however, remains controversial.^2^ Here, IR and other features of MetS were assessed in patients with moderate-to-severe AD.

This was a cross-sectional study conducted at the Hospital Universitario Ramón y Cajal (Madrid, Spain). The study included 50 consecutively recruited adult patients with moderate-to-severe AD (Eczema Area Severity Index [EASI] ≥12). Patients who had received systemic treatment within 4 weeks prior to inclusion were excluded. Thirty healthy controls were consecutively recruited from the same hospital. IR was determined using the homeostatic model assessment of insulin resistance (HOMA-IR), a quantitative index derived from fasting insulin and glucose. HOMA-IR was analyzed as a continuous variable and dichotomized using a cut-off value of 2.5 for certain sub-analyses. MetS was defined according to the harmonized International Diabetes Federation criteria. Methods are detailed in the Supplement, available via Mendeley at https://data.mendeley.com/datasets/9r6mmx3gvn/1.

Baseline characteristics are depicted in Table I. Patients with AD exhibited significantly higher IR, and a trend toward a higher prevalence of MetS compared to controls. In multivariable regression analysis, AD was associated with log-HOMA-IR (*β* = 0.24, *P* = .048) after adjusting for age, sex, waist circumference and current smoking (Supplementary Table II, available via Mendeley at https://data.mendeley.com/datasets/9r6mmx3gvn/1). When analyzing MetS components individually, patients with AD showed significantly higher triglycerides and waist circumference (Table I).

**Table I:** Demographic, clinical, and laboratory parameters in the atopic dermatitis and control groups

	AD	Controls	*P* value
	*N* = 50	*N* = 30	
Age, y	34.1 ± 12.4	39.8 ± 13.8	.061
Female, *n* (%)	14 (28)	9 (30)	.85
BMI, kg/m2	25.9 (22.2-31.9)	24.4 (22.2-26.0)	.17
Waist circumference, cm	92 (86-102)	87 (82-94)	**.045**
SBP, mm Hg	122.6 ± 13.8	116.8 ± 11.4	.057
DBP, mm Hg	79.6 ± 10.9	76.6 ± 8.5	.19
Current smokers, *n* (%)	18 (37)	5 (16.7)	.06
Total cholesterol, mg/dL	183.5 ± 36.1	188.5 ± 30.3	.53
LDL-cholesterol, mg/dL	112.2 ± 32.4	119.9 ± 28.3	.28
HDL-cholesterol, mg/dL	48 (39-58)	56.5 (44-61)	.068
Triglycerides, mg/dL	81 (66-100)	61 (47-83)	**.010**
Statin use, *n* (%)	3 (6)	2 (7)	.91
HOMA-IR	1.4 (1.2-2.1)	1.1 (0.8-1.4)	**.004**
Metabolic syndrome, *n* (%)	16 (32)	5 (17)	.13
EASI	22.1 (18.6-28.4)	-	-
BSA (%)	40 (30-55)	-	-
Disease duration, y	24 (14-32.5)	-	-
Hs-CRP, mg/dL	2.3 (1.4-4.3)	0.5 (0.4-1.4)	**<.001**
Previous systemic treatments, *n* (%)	38 (76)	-	-
Oral prednisone	36 (75)	-	-
Cyclosporine	8 (17)	-	-
Methotrexate	3 (6)	-	-
Phototherapy	8 (17)	-	-

Values reported in the table as mean ± standard deviation for normally distributed variables or median (interquartile interval) for non-normally distributed ones. Group comparisons were performed using *t*-test for normally distributed quantitative variables, Mann–Whitney *U* test for non-normally distributed variables, and chi-square tests for categorical variables.

Bold values indicate statistical significance.

*BMI*, Body mass index; *BSA*, body surface area; *DBP*, diastolic blood pressure; *EASI*, Eczema Area Severity Index; *HDL*, high-density lipoprotein; *HOMA-IR*, homeostatic model for assessment of insulin resistance, calculated as (fasting insulin [mU/ml] × fasting glucose [mg/dL])/405; *Hs-CRP*, high-sensitivity C-reactive protein; *LDL*, low-density lipoprotein; *SBP*, systolic blood pressure.

When exploring disease-related predictors of IR and MetS in AD patients (Supplementary Table III, available via Mendeley at https://data.mendeley.com/datasets/9r6mmx3gvn/1), those with MetS exhibited a higher number of prior ≥1-week oral corticosteroid courses (median 4.5 vs 2, *P* = .03) than those without MetS. The number of oral corticosteroid courses was associated with MetS adjusting for age, sex, EASI, and high-sensitivity C-reactive protein levels (OR 1.51 per course, 95% CI 1.10-2.09, Supplementary Table IV, available via Mendeley at https://data.mendeley.com/datasets/9r6mmx3gvn/1). Adjusted predicted probabilities of MetS according to oral corticosteroid courses were estimated using marginal predictions (Fig 1). These prior oral corticosteroid courses were also higher in those patients with HOMA-IR ≥2.5 (median 6 vs 2, *P* = .05), and correlated with BMI (*rho* = 0.38, *P* = .007), waist circumference (*rho* = 0.30, *P* = .04), and triglycerides (*rho* = 0.45, *P* = .001). EASI was inversely correlated with HDL-cholesterol (*rho* = −0.33, *P* = .02).

**Fig 1:**
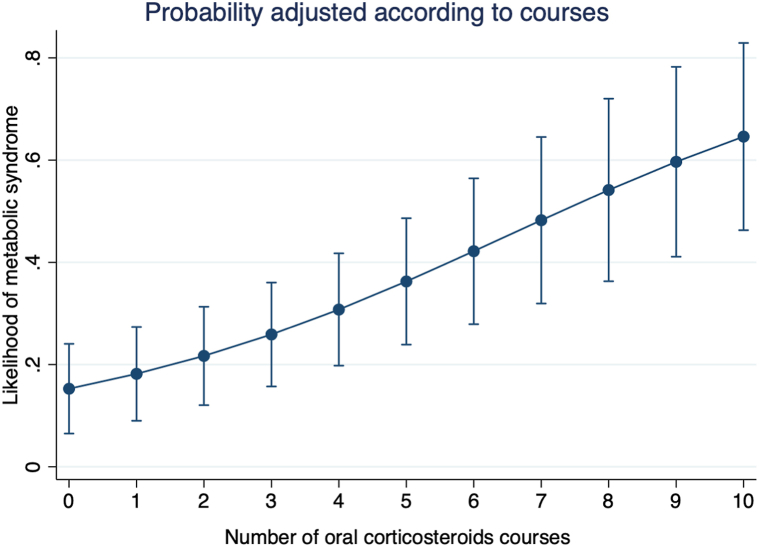
Adjusted predicted probability of metabolic syndrome according to the number of oral corticosteroid courses. Predicted probabilities were estimated from a multivariable logistic regression model adjusted for age, sex, EASI score, and high sensitivity C-reactive proteins levels. *EASI*, Eczema Area Severity Index.

These findings suggest that moderate-to-severe AD is associated with increased IR and dyslipidemia related to MetS. These results contrast with a previous study where no differences in IR were observed between AD patients and healthy controls.^3^ Of note, that study included AD patients who exhibited predominately mild-to-moderate disease (mean EASI 8.5), considerably lower than the cohort reported here. Disease severity likely plays a role in modulating metabolic co-morbidities. Moderate-to-severe AD is associated with systemic inflammation, which has been linked to IR in other chronic inflammatory diseases.^4^ Furthermore, cyclosporine and systemic corticosteroids, frequently employed in these patients, have also been associated with metabolic disturbances.^5^ Interestingly, in the study by *Shalom et al,* while patients with AD had an overall lower risk of MetS compared to controls, severity-stratified analysis demonstrated that patients with moderate-to-severe AD independently associated with higher MetS prevalence.^3^ Our results align with these findings, demonstrating that recurrent oral corticosteroid courses and disease severity are associated with several MetS components.

This report has limitations. A limited number of patients were studied from a single center, limiting the generalizability. The cross-sectional study design precludes directionality assessment. Finally, although HOMA-IR is a validated surrogate marker of IR, the euglycemic hyperinsulinemic clamp remains the gold-standard.

In conclusion, severe AD was associated with IR and several other MetS components. Prior oral corticosteroid use may increase this risk. These data could guide screening for IR and dyslipidemia in patients with moderate-to-severe AD, particularly those with higher cumulative systemic corticosteroid exposure.

### Declaration of generative AI and AI-assisted technologies in the writing process

This document used ChatGPT solely for grammar, spelling, and style corrections. The author reviewed and edited everything manually and takes full responsibility.

## Conflicts of interest

Dr Rico has potential conflict of interests (honorary for speaking and consultant) with the following pharmaceutical companies: Amgen, Leo Pharma, UCB, Abbvie and Viatris. Dr Mehta was a full-time US government employee and has served as a consultant for several pharmaceutical companies, receiving grants and/or research funding; and as a principal investigator for the NIH receiving grants and/or research funding. Dr Gelfand served as a consultant for Abbvie, Artax (DSMB), BMS, Boehringer Ingelheim, Celldex (DSMB), FIDE (which is sponsored by multiple pharmaceutical companies) GSK, Inmagene (DSMB), Lilly, Leo, Moonlake (DSMB), Janssen Biologics, Novartis Corp, UCB (DSMB), Neuroderm (DSMB), Oruka, and Veolia North America receiving honoraria; and receives research grants (to the Trustees of the University of Pennsylvania) from Amgen, BMS, and Pfizer Inc; and received payment for continuing medical education work related to psoriasis that was supported indirectly pharmaceutical sponsors. He is a co-patent holder of resiquimod for treatment of cutaneous T cell lymphoma. He is a Deputy Editor for the Journal of Investigative Dermatology receiving honoraria from the Society for Investigative Dermatology, is Chief Medical Editor for Healio Dermatology (receiving honoraria) and is a member of the Board of Directors for the International Psoriasis Council and the Medical Dermatology Society, receiving no honoraria. Dr Aranda was supported by a Marie Sklodowska-Curie Actions (MSCA) Postdoctoral Fellowships from Horizon Europe (101105416). Dr Blauvelt has served as a speaker (received honoraria) for Eli Lilly and Company and UCB, has served as a scientific adviser (received honoraria) for AbbVie, Almirall, Alumis, Amgen, Anaptysbio, Apogee, Arcutis, Boehringer Ingelheim, Bristol Myers Squibb, Celltrion, Corvus, Dermavant, Eli Lilly and Company, Galderma, GlaxoSmithKline, Immunovant, Incyte, IQVIA, Janssen, Leo, Lipidio, Merck, Novartis, Oruka, Paragon, Pfizer, Regeneron, Sanofi, Spherix Global Insights, Sun Pharma, Syncona, Takeda, UCB, and Union, has acted as a clinical study investigator (institution has received clinical study funds) for AbbVie, Acelyrin, Almirall, Alumis, Amgen, Arcutis, Boehringer Ingelheim, Bristol-Myers Squibb, Dermavant, Eli Lilly and Company, Galderma, Incyte, Janssen, Leo, Merck, Novartis, Pfizer, Regeneron, Sanofi, Sun Pharma, Takeda, and UCB, and owns stock in Lipidio and Oruka. Dr Gonzalez-Cantero has served as a consultant for Abbvie, Janssen, Novartis, Lilly, Almirall, BMS, Amgen, Boehringer Ingelheim, UCB, Celgene, and Leo Pharma receiving grants/other payments. Drs Garcia, Fuente, Bootello, Neria, Monge, Lopez, Jaen, and Author Aragon have no conflicts of interest to declare.
